# Left atrioventricular coupling index assessed by cardiac CT as a predictive marker for atrial fibrillation recurrence after ablation

**DOI:** 10.3389/fcvm.2026.1722711

**Published:** 2026-03-17

**Authors:** Guoxiang Du, Guoliang Lin, Hai Liao

**Affiliations:** 1 Department of Nuclear Medicine, Guangxi Medical University Cancer Hospital, Nanning, Guangxi, China; 2 Department of Radiology, the Second Affiliated Hospital of Guangxi Medical University, Nanning, Guangxi, China; 3 Department of Radiology, Gangkou District People’s Hospital of Fangchenggang, Fangchenggang, Guangxi, China

**Keywords:** atrial fibrillation ablation, cardiac CT, left atrioventricular coupling index, left ventricular volume index, recurrence

## Abstract

**Background:**

The left atrioventricular coupling index (LACI) is a significant predictor of cardiovascular events, heart failure, and atrial fibrillation (AF). However, its predictive efficacy for AF recurrence after ablation remains underexplored.

**Objectives:**

This study aimed to evaluate the predictive value of LACI, assessed by cardiac computed tomography (CT), for AF recurrence following ablation.

**Methods:**

We performed a retrospective analysis of 130 AF patients who underwent cardiac CT before ablation at our institution from January 2019 to March 2021. LACI, defined as the ratio of left atrial to left ventricular volume during diastole, was analyzed using multivariate binary logistic regression to determine its association with AF recurrence. Kaplan–Meier survival curves assessed the duration of asymptomatic survival post-ablation.

**Results:**

At 18 months, 29.2% (38/130) of patients experienced recurrence. Patients with recurrence had significantly higher LACI compared to those without (129.56 ± 48.02% vs. 84.58 ± 28.78%, *P* < 0.001). Multivariate analysis identified LACI as an independent risk factor for recurrence (OR = 9.51, 95% CI 1.99–45.34, *P* = 0.005), showing superior predictive capacity compared to the left atrial volume index (AUC: 0.815 vs. 0.779, *P* < 0.001). Kaplan–Meier curves demonstrated significantly lower cumulative AF recurrence-free survival in patients with LACI ≥ 89%.

**Conclusion:**

LACI is a strong and independent predictor of AF recurrence post-ablation, offering superior risk discrimination over traditional left atrial and left ventricular parameters, thereby enhancing the clinical utility of cardiac CT before AF ablation.

## Highlights

Independent Predictive Value of LACI: The left atrioventricular coupling index (LACI) is identified as an independent predictor of atrial fibrillation (AF) recurrence following catheter ablation, thereby offering novel insights for clinical risk stratification.
Superiority in Risk Discrimination: Compared with traditional single-chamber parameters of the left atrium or left ventricle, LACI demonstrates superior performance in risk discrimination, potentially enhancing the precision of AF recurrence prediction.
Prognostic Threshold of LACI: Patients with LACI ≥ 89% exhibit a significantly lower cumulative AF recurrence-free survival rate than those with LACI < 89%, suggesting that LACI may serve as a crucial biomarker for stratifying AF recurrence risk.

## 1 Introduction

Atrial fibrillation (AF) is one of the most common types of cardiac arrhythmias, with an overall prevalence of about 1%–2% in the general population (1). Its incidence increases with age and can reach 6.4% in people older than 80 years old (2). AF can significantly increase the risk of stroke, heart failure, and even sudden death, thus severely affecting the quality of life. Catheter ablation based on pulmonary venous isolation is an effective treatment method for patients with AF refractory to anti-arrhythmic drugs (3, 4). However, approximately 30% of patients with paroxysmal AF develop recurrence, and even more patients with persistent AF have multiple recurrences after ablation (5). Early identification of these high-risk groups is important. Although numerous risk scoring models exist in clinical practice for predicting the recurrence of AF, there remains a need for enhancement in their predictive capabilities (6).

Numerous studies have elucidated the intricate relationship between left atrial structural and functional remodeling and the recurrence of AF in patients following ablation procedures (7–9). Specifically, parameters such as left atrial morphology, size, function, volume index, and deformation are significant independent predictors of AF recurrence after ablation (10–13). However, relying solely on left atrial characteristics to assess the risk of AF recurrence has inherent limitations. Recent research has demonstrated that although left atrial size and deformation indices offer valuable insights, they may not encompass all factors contributing to AF recurrence. For instance, left ventricular diastolic dysfunction exerts a significant impact on both the recurrence rates of AF after ablation and the overall function of the left atrium (14, 15). This interconnection underscores the necessity for a more integrated approach when evaluating patients at risk of AF recurrence. In this context, the left atrioventricular coupling index (LACI), derived from magnetic resonance imaging measurements, has been recognized as a novel biomarker. It reflects left ventricular dysfunction more comprehensively than traditional left atrial measures (16, 17). Recent studies indicate that LACI is a superior predictor of AF occurrence compared to standard left atrial parameters, highlighting its potential clinical value in risk stratification (18). Moreover, the prognostic significance of LACI extends beyond AF prediction. It has been demonstrated that LACI provides enhanced prognostic value for heart failure outcomes and cardiovascular mortality compared to isolated left atrial and left ventricular parameters (19, 20). Furthermore, emerging evidence suggests that LACI, measured by cardiac computed tomography (CT), has an independent correlation with the incidence of cardiovascular events, indicating its increasing prognostic relevance (21). The unique advantage of LACI lies in its capacity to integrate information on both atrial and ventricular dynamics, thereby providing a more comprehensive assessment of the patient's cardiovascular status and future risk of adverse events.

This study aimed to determine the prognostic value of LACI based on cardiac CT measurements for the recurrence of AF after ablation, thereby expanding the application value of cardiac CT before atrial fibrillation ablation.

## 2 Materials and methods

### 2.1 Materials

**Figure 1:**
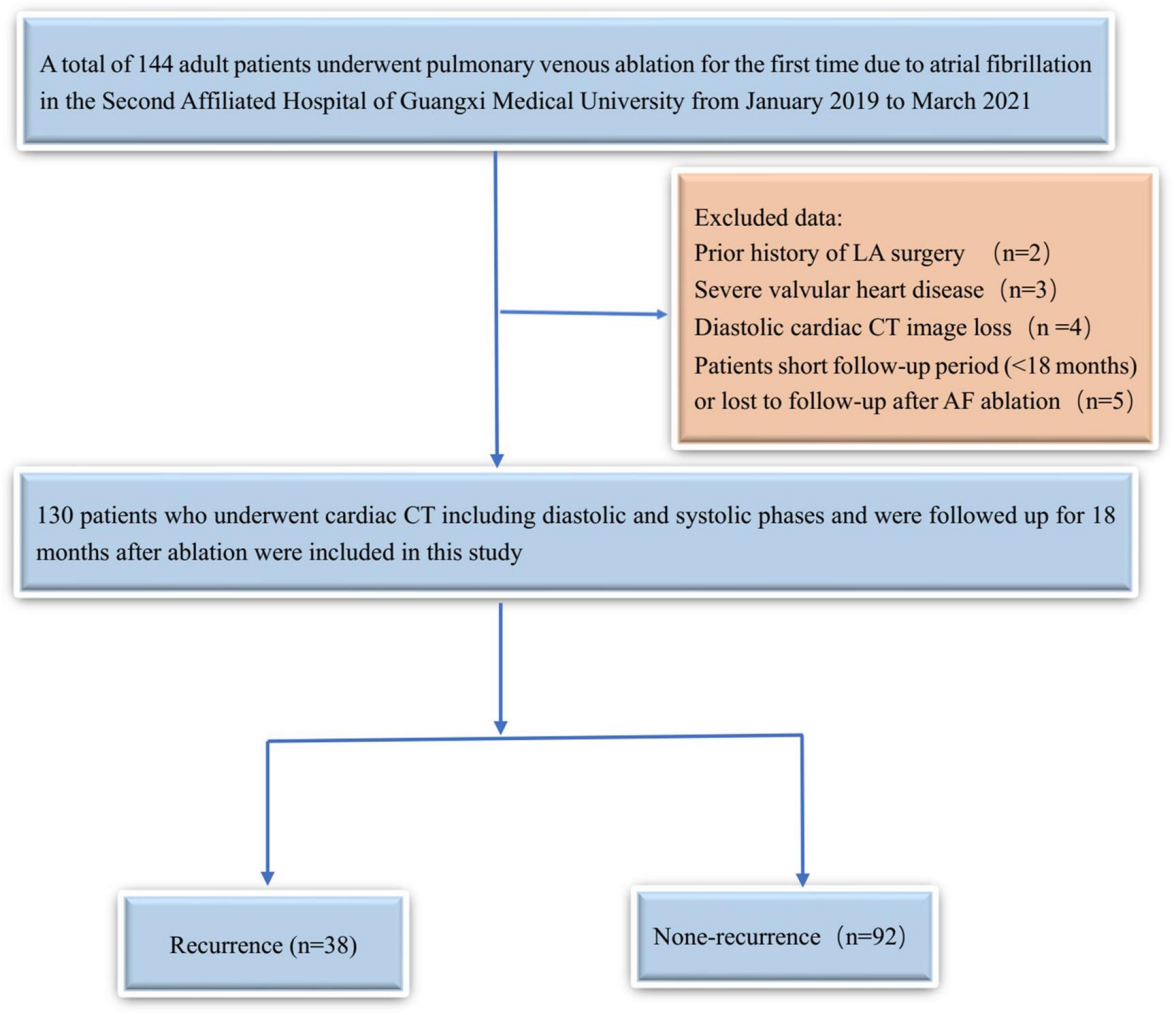
The patient enrollment flow diagram.

This study retrospectively analyzed patients who underwent pulmonary venous ablation for the first time due to AF at the Second Affiliated Hospital of Guangxi Medical University between January 2019 and March 2021 (Figure 1). The inclusion criteria were as follows: (1) patients with a previous electrocardiogram (ECG) or 24-hour ECG diagnosis of AF; (2) patients aged 18 years or older; and (3) patients who underwent cardiac CT examination within 7 days prior to ablation. The exclusion criteria were as follows: (1) patients with a prior history of left atrial surgery and ablation; (2) patients post-pacemaker implantation; (3) patients with severe valvular heart disease; (4) patients with poor-quality, missing, or incomplete CT images (which must include optimal diastolic and systolic phases); and (5) patients lacking follow-up. For the purposes of this retrospective analysis, we collected clinical data from the patients, including age, gender, body mass index (BMI), blood pressure, heart rate, type of AF, history of hypertension, diabetes, smoking status, alcohol consumption, method of ablation, and whether there was a prior history of AF episodes.

### 2.2 CT acquisition protocol

All patients provided informed consent and underwent cardiac CT within 7 days prior to ablation for anatomical evaluation of the left atrium and pulmonary veins, as well as for procedural planning and mapping, they were scanned from the aortic arch to the base of the heart using a Philips Brilliance 256-slice iCT. Cardiac CT was performed in retrospective electrocardiogram-gated mode with a tube voltage of 100 kV. For patients with a body mass index (BMI) ≥ 25 kg/m^2^, the tube current was set to 350 mAs, while for those with a BMI <25 kg/m^2^, the tube current was set to 250 mAs. For patients with elevated heart rate, particularly those with persistent atrial fibrillation, oral beta-blockers (metoprolol, 25 mg per tablet) were administered before cardiac CT to achieve a ventricular rate below 80 beats per minute and optimize imaging of the left atrium and pulmonary veins. A nonionic contrast agent (350 mg I/mL iodohexol) was injected using a double-barreled high-pressure syringe at a flow rate of 5 mL/s, followed by 30 mL of normal saline at the same rate. The region of interest (ROI) was delineated at the tracheal bifurcation of the descending aorta using contrast tracking and triggering techniques, with a triggering threshold of 100 HU. The acquired images were processed using the Philips post-processing workstation.

### 2.3 Image processing

**Figure 2:**
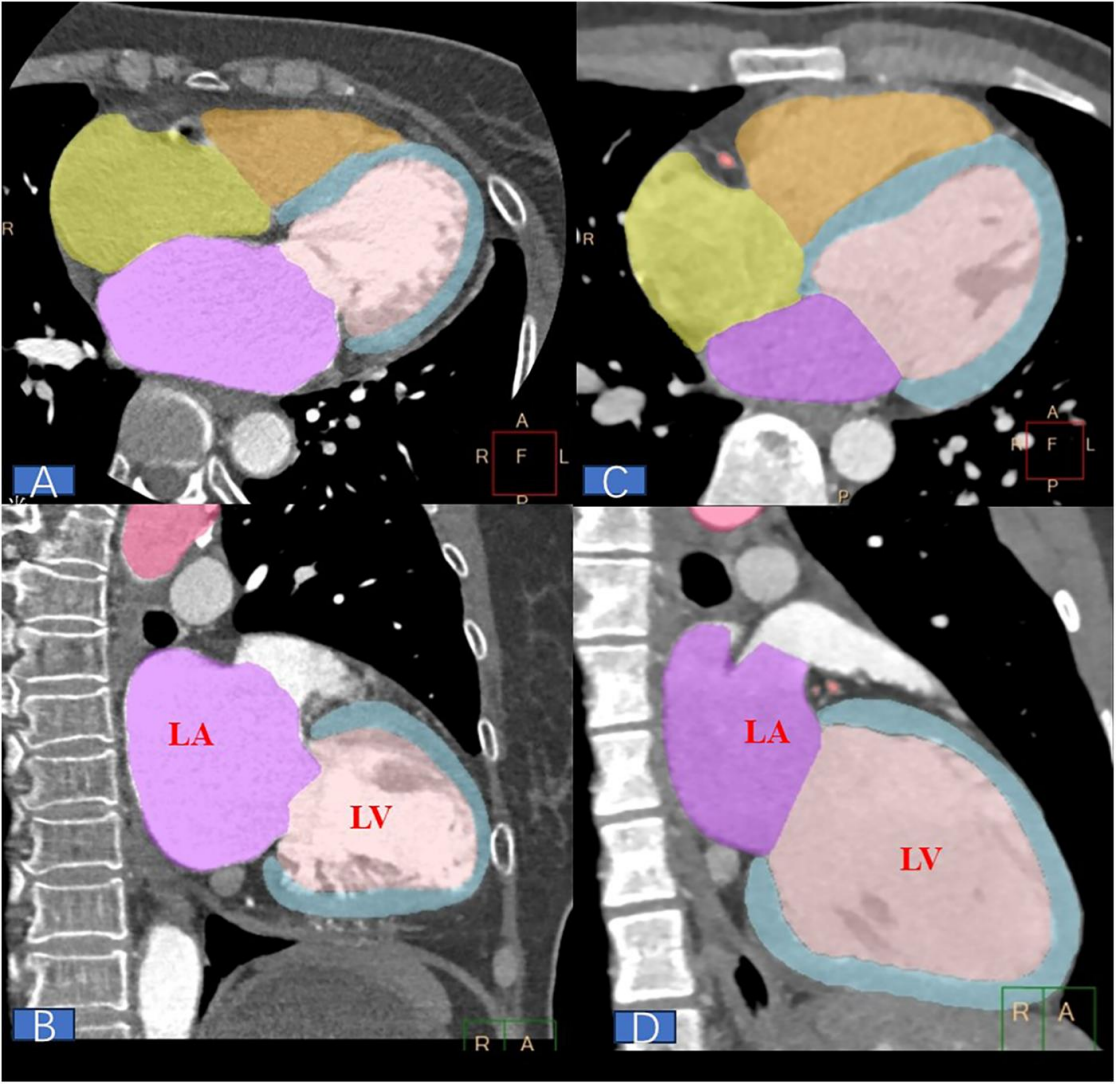
**(A,C)** shows the best apical four-chamber view during diastole, while B and D illustrate the best apical long-axis view of the left ventricle during diastole. LACI is defined as the ratio of the left atrial volume to the left ventricular volume at the best diastolic phase. **(A,B)** depict a 68-year-old female patient with persistent atrial fibrillation, who had a LACI of 214% and experienced recurrence 97 days after ablation. In contrast, C and D depict a 22-year-old male patient with paroxysmal atrial fibrillation, who had a LACI of 39% and showed no recurrence during the 540-day follow-up after ablation.

The optimal systolic and diastolic phases of the original images were reconstructed and transmitted to the Philips Syngovia VB10B workstation for cardiac function analysis. The workstation automatically outlined the endocardial margins of the left atrial volume index (LAVI), right atrial volume index (RAVI), left ventricular volume index (LVVI), and right ventricular volume index (RVVI). If the endocardial margins were deemed unsatisfactory, manual adjustments were undertaken. According to Simpson's formula, the diastolic and systolic volume indices of both atria and ventricles were automatically calculated. All data were independently measured by two radiologists with over 10 years of experience: GX. Du and GL. Lin. LACI was defined as the ratio of left atrial volume to left ventricular volume during the optimal diastolic phase, as illustrated in Figure 2. In patients with persistent atrial fibrillation, the accuracy of left atrial and ventricular measurements during CT scans may be compromised. To mitigate this issue, we employed a retrospective multi-phase reconstruction technique to measure the maximum and minimum volume indices of the left atrium and ventricle. In this context, the left atrial-ventricular coupling index is defined as the ratio of the maximum volume of the left atrium to the minimum volume of the left ventricle, thereby minimizing measurement errors.

### 2.4 Pulmonary vein ablation methods for AF

The selection of cryoablation or radiofrequency ablation was based on the 2020 ESC/EHRA Atrial Fibrillation Guidelines and individual patient characteristics, including atrial fibrillation type, left atrial anatomy, and procedural complexity (22).41 patients underwent cryoablation, while 89 patients underwent radiofrequency ablation. The ablation procedures were conducted under local anesthesia. Radiofrequency ablation was performed under the guidance of the CARTO system for three-dimensional electrical mapping of the left atrium and pulmonary veins, as well as for anatomical mapping and circumferential pulmonary vein isolation. Cryoballoon ablation was performed for pulmonary vein isolation (PVAI) using an Arctic Front Advance Catheter (Medtronic) and an Achieve Mapping Catheter (Medtronic). After vestibular aeration, distal pulmonary vein angiography was performed to confirm the absence of contrast agent leakage, followed by vestibular freezing. Successful ablation is characterized by the gradual extinction of left and right pulmonary vein potentials until they are no longer detectable, or by the persistence of AF rhythm post-ablation, which can be successfully reverted to sinus rhythm through direct current cardioversion after intravenous diazepam injection. All patients in this study were successfully ablated without complications.

### 2.5 Postoperative follow-up

Telephone or outpatient follow-up was performed by trained specialized staff after the ablation. The follow-up included a standard 12-lead electrocardiogram and 24-hour Holter ECG at 3,6,12, and 18 months post-ablation. Additionally, if a patient developed palpitations or other symptoms suggestive of arrhythmia, an ECG was performed at a local hospital at any time, and the ECG was transmitted to the follow-up center for interpretation by the follow-up personnel. Recurrence was defined as any documented atrial tachyarrhythmia (AF, atrial flutter, or atrial tachycardia) recorded by ECG or Holter monitoring lasting more than 30 s after a 3-month blanking period. Except for patients with recurrence, all patients were followed for at least 18 months after ablation.

### 2.6 Statistical analysis

SPSS 22 statistical analysis software was utilized. Measurement data conforming to normal distribution were expressed as mean ± standard deviation, while those not conforming to normal distribution were expressed as median (upper and lower quartiles). The normality of data was evaluated using the Shapiro–Wilk test, an independent sample t-test was employed to compare differences in LACI, LAVI, RAVI, LVVI, RVVI, BMI, blood pressure, and heart rate between recurrence and non-recurrence groups after AF ablation. The Chi-square test was applied for gender, type of AF, hypertension, diabetes, smoking, and alcohol consumption. The consistency of the LACI was evaluated using the intraclass correlation coefficient (ICC). Multivariate binary logistic regression was employed to analyze risk factors associated with AF recurrence post-ablation. Receiver operating characteristic (ROC) curves were constructed to analyze the diagnostic efficacy of each parameter in predicting AF recurrence and to determine the clinical cut-off value, The optimal cut-off value was determined by the Youden index. The area under the curve (AUC) was calculated and reported. Kaplan–Meier survival curves and log-rank tests were utilized to analyze the recurrence-free survival rate of AF during the 18-month follow-up period post-ablation. A two-sided *P*-value of < 0.05 was considered statistically significant.

## 3 Results

### 3.1 Patient general information

Among 144 patients who underwent pulmonary vein ablation for the first time, 130 fulfilled the inclusion criteria and were retrospectively enrolled in the analysis. Of these 130 patients, 73 (56.15%) had paroxysmal atrial fibrillation and 57 (43.85%) had persistent atrial fibrillation. Cryoablation was performed in 41 patients (31.54%), while radiofrequency ablation was performed in 89 patients (68.46%). There were 78 male patients (60%) and 52 female patients (40%), with a mean age of 63.4 ± 11.8 years. During the 18-month follow-up after AF ablation, 29.2% (38/130) of patients experienced recurrence, and detailed baseline characteristics are presented in Tables 1, 2. The LACI measurements obtained by the two radiologists showed good reproducibility, with an ICC of 0.99 (*P* < 0.001).

**Table 1:** Pair t-test of recurrence group and non-recurrence group after ablation.

Parameters	All cases (*n* = 130)	recurrence (*n* = 38)	non-recurrence (*n* = 92)	*T*-value	*P*-value
LACI（%）	97.73 ± 40.83	129.56 ± 48.02	84.58 ± 28.78	−5.38	<0.001
LAVI (mL/m^2^)	45.44 ± 18.91	58.81 ± 23.12	39.93 ± 12.56	−4.71	<0.001
RAVI (mL/m^2^)	46.31 ± 18.19	54.44 ± 20.15	42.95 ± 16.27	−3.12	0.003
LVVI （mL/m^2^）	41.23 ± 13.15	42.41 ± 15.47	40.75 ± 12.13	−0.65	0.52
RVVI（mL/m^2^）	46.65 ± 12.74	47.16 ± 13.83	46.45 ± 12.34	−0.28	0.77
LVEF（%）	48.11 ± 17.73	41.98 ± 19.53	50.63 ± 16.38	2.58	0.011
BMI （kg/m^2^）	24.53 ± 4.23	24.29 ± 4.07	24.62 ± 4.31	0.40	0.68
Blood Pressure （mmHg）	128.16 ± 17.16	125.74 ± 18.49	129.16 ± 16.58	1.03	0.32
Heart Rate (bpm)	83.83 ± 17.77	83.03 ± 16.19	84.16 ± 18.46	0.34	0.72
Age (y)	63.44 ± 11.87	68.39 ± 9.53	61.39 ± 12.18	−3.50	0.002

BMI, body mass index; LACI, left atrioventricular coupling index; LAVI, left atrial volume index; RAVI, right atrial volume index; LVVI, left ventricular volume index; RVVI, right ventricular volume index; LVEF, left ventricular ejection fraction.

**Table 2:** Chi-square test for recurrence vs. non-recurrence after ablation.

Characteristics	All cases (*n* = 130)	Recurrence（*n* = 38)	non-recurrence（*n* = 92)	*χ*^2^ value	*P*-value
Gender (Male%)	78 (60%)	16（42.11%）	62（67.39%）	7.16	0.007
AF type (paroxysmal%)	73 (56.2%)	15（39.47%）	58（63.04%）	6.06	0.014
Hypertension (%)	71 (54.6%)	21（55.26%）	50（54.34%）	0.09	0.92
Diabetes (%)	20 (15.4%)	9（23.68%）	11（11.95%）	2.84	0.09
Smoking (%)	43 (33.1%)	11（28.94%）	32（34.78%）	0.41	0.52
Alcohol consumption (%)	45 (34.6%)	14（36.84%）	31（33.69%）	0.11	0.73
Ablation method（cryoablation%）	41 (31.53%)	12（31.57%）	29（31.52%）	0.84	0.65

AF, atrial fibrillation.

### 3.2 Comparison of patients with and without recurrent AF after ablation

Patients with recurrence after ablation were significantly older and had higher levels of LACI, LAVI, and RAVI compared with those without recurrence (LACI 129.56 ± 48.02% vs. 84.58 ± 28.78%, *P* < 0.001; LAVI 58.81 ± 23.12 mL/m^2^ vs. 39.93 ± 12.56 mL/m^2^, *P* < 0.001). There were significant differences in gender and AF type between groups, Specifically, the proportion of males was significantly lower in the recurrence group than in the non-recurrence group (42.11% vs. 67.39%, *χ*^2^ = 7.16, *P* = 0.007). Additionally, the proportion of paroxysmal AF was significantly lower in the recurrence group than in the non-recurrence group (39.47% vs. 63.04%, *χ*^2^ = 6.06, *P* = 0.014). No significant differences were found in LVVI, RVVI, BMI, systolic blood pressure, heart rate, hypertension, diabetes, smoking, ablation method and alcohol drinking consumption among the groups (*P* > 0.05), as shown in Tables 1, 2.

### 3.3 Predictive value of LACI for AF recurrence after catheter ablation

**Figure 3:**
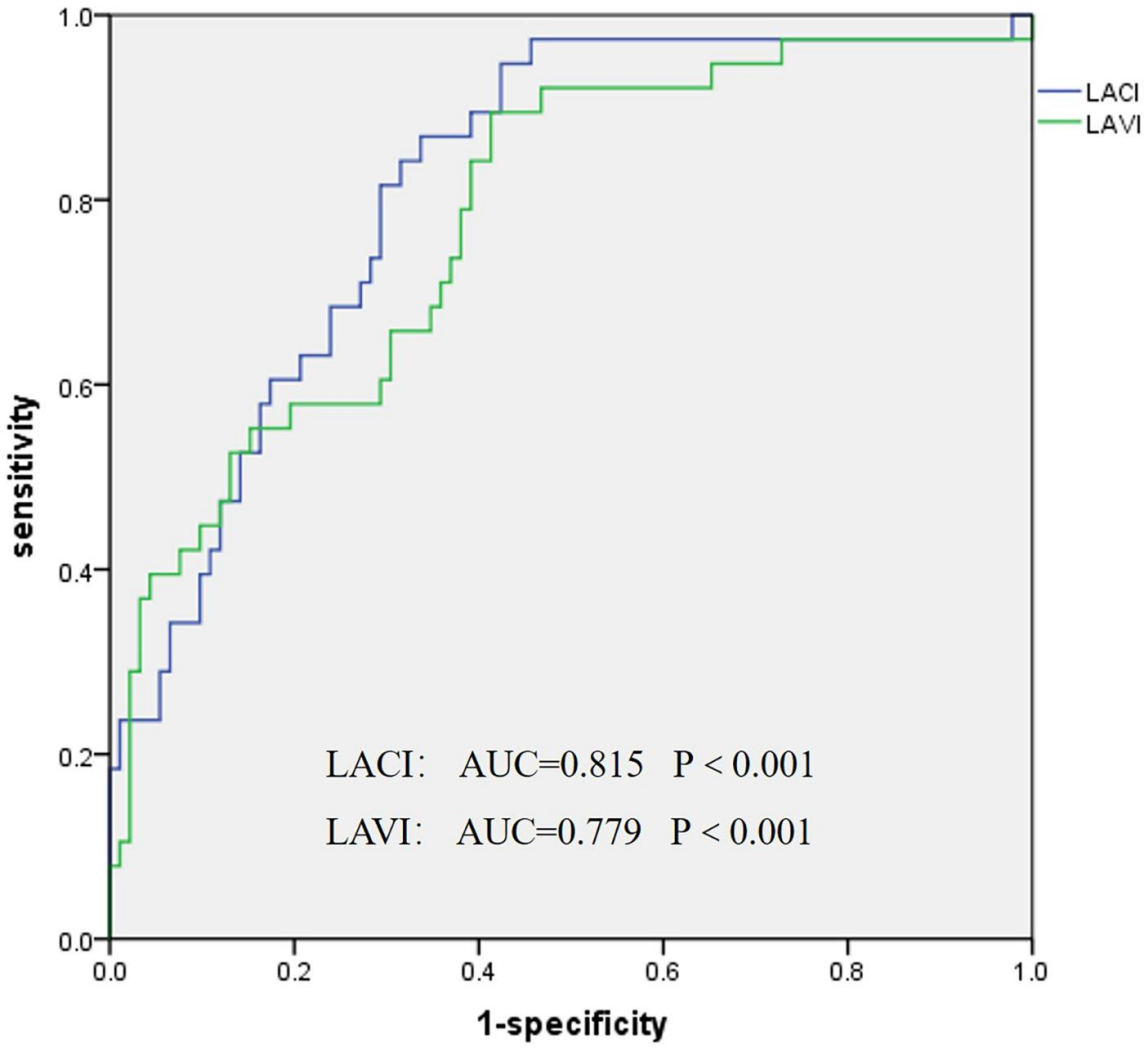
Receiver operating characteristic curve of the left atrioventricular coupling index and left atrial volume for predicting recurrence after ablation.

After ablation, the statistically significant parameters of recurrence and non-recurrence were included in multivariate binary logistic regression analysis. LACI was the only independent risk factor for predicting the recurrence of AF (LACI OR = 9.51, 95% CI 1.99–45.34, *P* = 0.005), as shown in Table 3. LACI was superior to LAVI in predicting the recurrence of AF after ablation: the AUC was 0.815 vs. 0.779, *P* < 0.001; 95% confidence interval 0.737–0.892 vs. 0.691–0.866; the sensitivity was 86.8% vs. 89.5%, the specificity was 66.3% vs. 58.7%, and the LACI cut-off value was 89% (Figure 3).

**Table 3:** Risk factors for predicting recurrence of AF by multivariate binary logistic regression analysis.

Parameters	All cases (*n* = 130)			Paroxysmal AF (*n* = 73)			Persistent AF (*n* = 57)		
	OR value	95%CI	*P-*value	OR value	95%CI	*P* value	OR value	95%CI	*P-*value
LACI（%）	9.51	1.99–45.34	0.005	4.38	0.17–112.73	0.37	23.86	2.55–223.16	0.005
LAVI (mL/m^2^)	1.04	0.99–1.11	0.07	1.02	0.93–1.13	0.57	1.04	0.99–1.09	0.08
RAVI (mL/m^2^)	0.98	0.94–1.03	0.60	1.01	0.94–1.08	0.85			
LVEF（%）	0.32	0.02–5.99	0.45						
Age（y）	1.03	0.97–1.08	0.34	1.04	0.97–1.12	0.22	1.00	0.92–1.08	0.95
Gender (M%)	0.90	0.33–2.49	0.84				0.66	0.15–2.96	0.59
AF type (paroxysmal%)	0.99	0.33–3.00	0.46						

LACI, left atrioventricular coupling index; LAVI, left atrial volume index; RAVI, right atrial volume index; LVEF, left ventricular ejection fraction; AF, atrial fibrillation.

**Figure 4:**
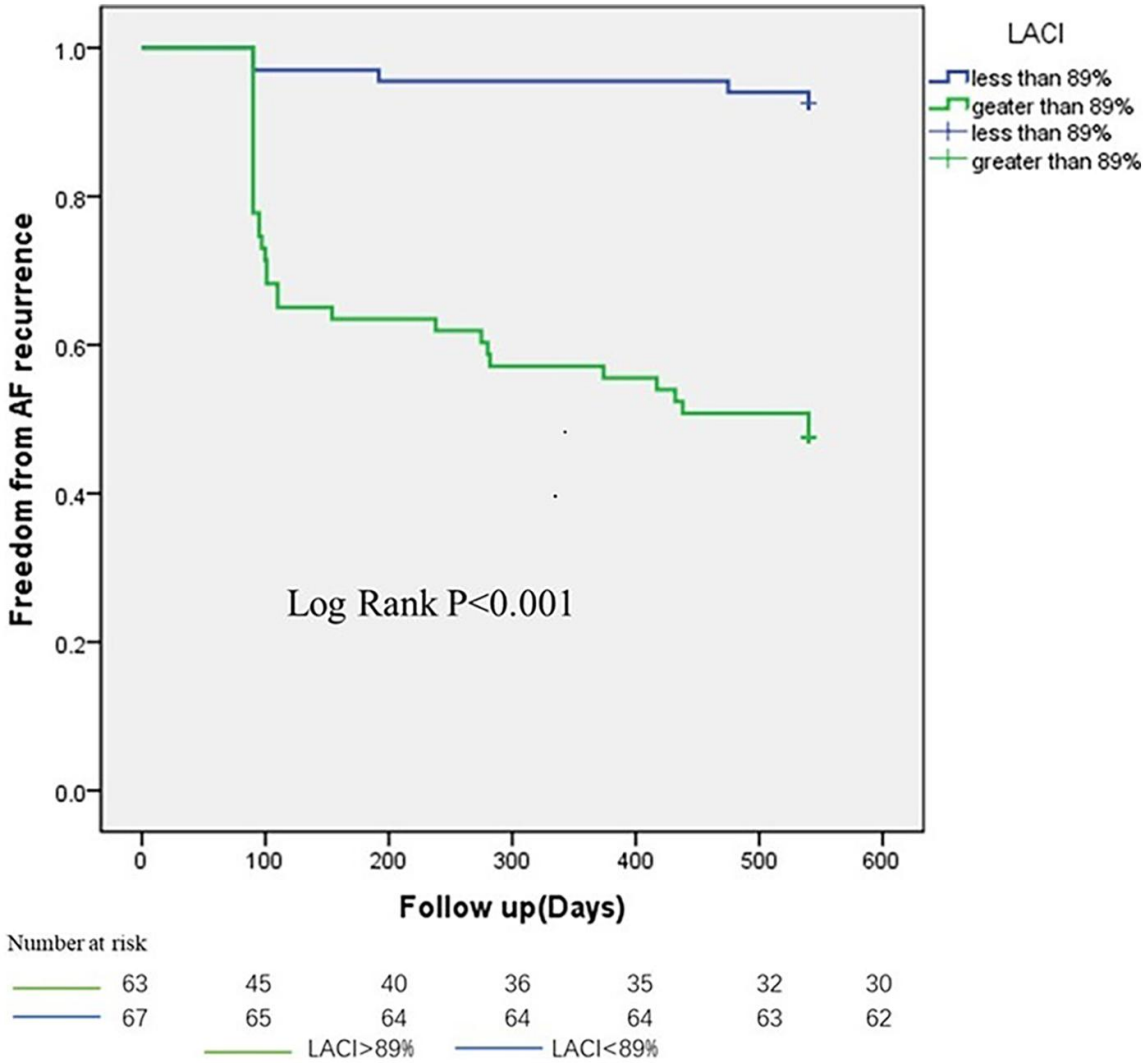
Kaplan–Meier survival curve analysis of Atrial fibrillation recurrence-free survival during 18-month follow-up after ablation.

Next, the data were divided into the LACI ≥ 89% group and the LACI < 89% group. Kaplan–Meier survival curve analysis showed that the cumulative recurrence-free survival rate of AF was significantly different between patients in the LACI group according to the LACI cut-off value. The cumulative recurrence-free survival rate of AF in patients with LACI ≥89% was significantly reduced (353 vs. 520 days, *P* < 0.001), as shown in Figure 4.

### 3.4 Subgroup analysis – LACI in paroxysmal AF for predicting recurrence after ablation

Among the 73 patients with paroxysmal AF, 15 (20.5%) experienced recurrence after 18 months of ablation follow-up. Compared with the non-recurrence group, LACI, LAVI, RAVI, and age in the recurrence group were significantly higher than those in the non-recurrence group, and the differences were statistically significant (LACI: 105.61 ± 35.56% vs. 78.55 ± 26.60%, *P* = 0.002; LAVI: 49.07 ± 16.06 mL/m^2^ vs. 36.13 ± 12.14 mL/m^2^, *P* = 0.001). There were no significant differences in LVVI, RVVI, BMI, systolic blood pressure, heart rate, gender, hypertension, diabetes, smoking, and drinking among groups (all *P* > 0.05) (Table 4).

**Table 4:** Pair t-test for recurrence group and non-recurrence group after ablation in patients with paroxysmal AF and persistent AF.

Parameters	paroxysmal AF (*n* = 73)				persistent AF (*n* = 57)			
	recurrence (*n* = 15)	non-recurrence (*n* = 58)	*T*-value	*P*-value	recurrence (*n* = 23)	non-recurrence (*n* = 34)	*T*-value	*P*-value
LACI（%）	105.61 ± 35.56	78.55 ± 26.60	−3.18	0.002	145.18 ± 49.27	94.87 ± 28.20	−4.43	<0.001
LAVI（mL/m^2^）	49.07 ± 16.06	36.13 ± 12.14	−3.43	0.001	65.15 ± 25.06	46.41 ± 13.58	−3.27	0.003
RAVI（mL/m^2^）	46.73 ± 14.01	38.05 ± 12.82	−2.17	0.042	59.47 ± 22.16	51.30 ± 18.23	−1.52	0.13
LVVI （mL/m^2^）	40.56 ± 9.17	38.62 ± 10.21	−0.67	0.50	43.61 ± 18.59	44.36 ± 14.29	0.17	0.86
RVVI （mL/m^2^）	44.79 ± 10.84	44.43 ± 9.92	−0.12	0.90	48.69 ± 15.52	49.89 ± 15.19	0.28	0.77
LVEF（%）	53.04 ± 11.41	54.89 ± 14.07	0.47	0.64	34.77 ± 20.52	43.36 ± 17.64	1.68	0.09
BMI （kg/m^2^）	23.24 ± 3.12	24.98 ± 3.61	1.69	0.04	24.98 ± 4.51	24.02 ± 5.30	−0.71	0.48
Blood Pressure（mmHg）	132.67 ± 14.59	130.55 ± 15.53	0.47	0.63	121.21 ± 19.64	126.79 ± 18.24	1.09	0.27
Heart Rate (bpm)	77.27 ± 15.25	82.52 ± 20.45	0.93	0.35	86.78 ± 15.99	86.97 ± 14.27	0.04	0.96
Age(y)	69.47 ± 11.95	60.69 ± 13.34	−2.31	0.02	67.70 ± 7.78	62.59 ± 9.95	−2.06	0.04

BMI, body mass index; LACI, left atrioventricular coupling index; LAVI, left atrial volume index; RAVI, right atrial volume index; LVVI, left ventricular volume index; RVVI, right ventricular volume index; LVEF, left ventricular ejection fraction.

Next, parameters with statistically significant differences between groups were included in the multivariate binary logistic regression analysis, and the results indicated that none of these factors served as independent risk factors for predicting AF recurrence, as shown in Table 3. Although LACI was not an independent predictor for predicting the recurrence of paroxysmal AF, it could still predict the recurrence of AF well, and AUC was 0.749, *P* = 0.003.

### 3.5 Subgroup analysis – LACI in persistent AF predicting recurrence after ablation

Among the 57 patients with persistent AF, 23 (40.4%) experienced recurrence during the 18-month follow-up after ablation. Compared with the non-recurrence group, the LACI, LAVI, and age in the recurrence group were significantly higher than those in the non-recurrence group, and the differences were statistically significant (LACI: 145.18 ± 49.27%vs.94.87 ± 28.20%, *P* < 0.001; LAVI: 65.15 ± 25.06 mL/m^2^ vs.46.41 ± 13.58 mL/m^2^, *P* < 0.001). Statistically significant differences were observed in gender between the two groups, while there were no significant differences in LVVI, RVVI, RAVI, BMI, systolic blood pressure, heart rate, hypertension, diabetes, smoking, and drinking among groups (*P* > 0.05) (Table 4). Multivariate binary logistic regression analysis included parameters with statistically significant differences between groups. The results showed that LACI was an independent risk factor for predicting recurrence after ablation in patients with persistent AF (LACI OR = 23.86, 95% CI 2.55–223.16, *P* = 0.005) and could well predict recurrence after ablation of AF, with an AUC of 0.824, *P* < 0.001, as shown in Table 3.

## 4 Discussion

In our study, it was found that LACI served as the sole independent predictor of recurrence following AF ablation (OR = 9.51, *P* = 0.005). Furthermore, LACI demonstrated superiority over individual left atrial or left ventricular parameters in identifying patients at high risk of recurrence after AF ablation. Notably, the relapse-free survival time for AF patients with LACI ≥ 89% was significantly reduced.

In this study, we defined LACI as the diastolic LA to LV volume ratio while excluding the possible influence of severe valvular disease and cardiomyopathy on the study results. During diastole, the LA is directly exposed to pressure in the LV, which elevates as LV diastolic function deteriorates; To maintain adequate ventricular filling, LA pressure increases accordingly, leading to elevated atrial wall tension. This, in turn, induces atrial electrical and structural remodeling—including myocardial stretch, dilatation, and fibrosis—all of which promote the initiation, maintenance, and recurrence of AF (23, 24). Recent studies have demonstrated that LA diastolic volume is more reliable than systolic volume in predicting cardiovascular events (25, 26). Olsen et al. (27) investigated the association between isometric and allometric height-indexed LA size and AF in healthy people, finding that isometric and allometric height-indexed LA volumes are associated with AF in the general population. LAV_min_ is more strongly associated with AF than LAV_max_, regardless of indexation. Furthermore, Kohári et al. (28) suggested diastolic LA volume as the only independent imaging parameter associated with AF recurrence in a study of risk factors for recurrence after ablation in patients with persistent and long-standing persistent AF. Therefore, the diastolic LACI can more objectively reflect the left atrial and ventricular remodeling in patients with AF and is superior to the traditional single left atrial and left ventricular parameters in predicting the recurrence of AF after ablation, which is consistent with our results. In addition, it should be noted that the retrospective CT protocol used in the present study to evaluate both systolic and diastolic phases is associated with increased radiation exposure. In contrast, a prospective CT protocol or cardiac magnetic resonance imaging (CMR) could reduce the radiation burden while providing comparable or even more comprehensive functional assessment.

More importantly, the prognostic value of the defined LACI cut-off of 89% is robustly validated by the finding that patients with LACI ≥ 89% had significantly shorter AF recurrence-free survival after catheter ablation (353 vs. 520 days, *P* < 0.001). Derived via the Youden index from ROC curve analysis with high inter-observer reproducibility (ICC > 0.99, *P* < 0.001), this 89% cutoff is substantially higher than the 34.5% and 35.5% thresholds reported in two echocardiography-based studies of paroxysmal AF patients (29, 30), attributable to our cohort including both paroxysmal and persistent AF patients with more severe left atrioventricular remodeling, as well as inherent imaging modality differences. All studies nonetheless confirm LACI as an independent predictor of post-ablation AF recurrence, and this 89% cutoff tailored to patients with advanced remodeling offers practical clinical value for preoperative risk stratification and personalized management, highlighting the need for modality- and AF subtype-specific LACI reference values.

We further analyzed paroxysmal AF and persistent AF in the subgroup of AF, finding that LACI was an independent risk factor for predicting recurrence after ablation in patients with persistent AF. LACI was a good predictor of recurrence after ablation, and the AUC was 0.824. Although LACI was not an independent predictor for predicting the recurrence of paroxysmal AF, it could still predict the recurrence of AF well, and the AUC was 0.749. This result may be because persistent AF increases left atrial and ventricular remodeling, and left ventricular diastolic function is significantly impaired, resulting in more advanced left atrial remodeling. However, patients with paroxysmal AF had shorter arrhythmia times and less influence on left atrial ventricular remodeling, which is consistent with that reported by Kohári et al. (28). The value of left atrioventricular coupling index in predicting cardiovascular and thromboembolic events was limited, which may be due to the small sample size and short follow-up duration, leading to potential bias in the results; this warrants further investigation in subsequent studies.

The present study has few limitations. First, this was a single-center retrospective observational study with relatively limited research volume and potential selection bias. Second, the gold standard for left atrioventricular volume measurement is MR. In this study, we used cardiac CT measurement. Cardiac CT measures left ventricular volumes roughly equivalent to MR; left atrial volumes were slightly overestimated, and left atrial ejection fraction was underestimated (31, 32). Third, the patients with persistent atrial fibrillation, CT scans are not conducted under sinus rhythm, which may affect the accuracy of left atrial and ventricular volume measurements. Finally, Holter monitoring may not be the optimal method for detecting atrial fibrillation recurrence, and future prospective studies should consider the use of implantable loop recorders (ILRs) instead.

In conclusion, the LACI serves as a robust and independent predictor of AF recurrence following ablation. Notably, it demonstrates superior risk discrimination capabilities compared to traditional parameters focused solely on the left atrium or left ventricle. This finding is instrumental in stratifying patients at high risk for recurrence after AF ablation, thereby enhancing the clinical utility of cardiac CT prior to the ablation.

## Data Availability

The original contributions presented in the study are included in the article/Supplementary Material, further inquiries can be directed to the corresponding author.
